# Changes in serial multiparametric MRI and FDG-PET/CT functional imaging during radiation therapy can predict treatment response in patients with head and neck cancer

**DOI:** 10.1007/s00330-023-09843-2

**Published:** 2023-07-05

**Authors:** Yuvnik Trada, Paul Keall, Michael Jameson, Daniel Moses, Peter Lin, Phillip Chlap, Lois Holloway, Myo Min, Dion Forstner, Allan Fowler, Mark T. Lee

**Affiliations:** 1 Department of Radiation Oncology, Calvary Mater Newcastle, Edith St, Waratah, NSW 2298 Australia; 2 Faculty of Medicine and Health, Sydney Medical School, The University of Sydney, Sydney, NSW Australia; 3 ACRF Image X Institute, University of Sydney, Sydney, NSW Australia; 4 GenesisCare St Vincents Hospital, Sydney, NSW Australia; 5 St Vincents Clinical School, Faculty of Medicine, University of Sydney, Sydney, NSW Australia; 6 Graduate School of Biomedical Engineering, Faculty of Engineering, University of New South Wales, Sydney, NSW Australia; 7 Department of Medical Imaging, Prince of Wales Hospital, Randwick, NSW Australia; 8 Department of Nuclear Medicine and PET, Liverpool Hospital, Liverpool, NSW Australia; 9 School of Medicine, Western Sydney University, Sydney, NSW Australia; 10 Department of Radiation Oncology, Cancer Therapy Centre, Liverpool Hospital, Liverpool, NSW Australia; 11 South Western Clinical School, School of Medicine, University of New South Wales, Sydney, NSW Australia; 12 Ingham Institute of Applied Medical Research, Liverpool, NSW Australia; 13 University of Sunshine Coast, Birtinya, QLD Australia; 14 Sunshine Coast University Hospital, Sunshine Coast, QLD Australia; 15 Griffith University, Sunshine Coast, QLD Australia

**Keywords:** Biomarker, Radiotherapy, Head and neck neoplasms, Magnetic resonance imaging, Fluorodeoxyglucose F18

## Abstract

**Objectives:**

To test if tumour changes measured using combination of diffusion-weighted imaging (DWI) MRI and FDG-PET/CT performed serially during radiotherapy (RT) in mucosal head and neck carcinoma can predict treatment response.

**Methods:**

Fifty-five patients from two prospective imaging biomarker studies were analysed. FDG-PET/CT was performed at baseline, during RT (week 3), and post RT (3 months). DWI was performed at baseline, during RT (weeks 2, 3, 5, 6), and post RT (1 and 3 months). The ADC_mean_ from DWI and FDG-PET parameters SUV_max_, SUV_mean_, metabolic tumour volume (MTV), and total lesion glycolysis (TLG) were measured. Absolute and relative change (%∆) in DWI and PET parameters were correlated to 1-year local recurrence. Patients were categorised into favourable, mixed, and unfavourable imaging response using optimal cut-off (OC) values of DWI and FDG-PET parameters and correlated to local control.

**Results:**

The 1-year local, regional, and distant recurrence rates were 18.2% (10/55), 7.3% (4/55), and 12.7% (7/55), respectively. ∆Week 3 ADC_mean_ (AUC 0.825, *p* = 0.003; OC ∆ > 24.4%) and ∆MTV (AUC 0.833, *p* = 0.001; OC ∆ > 50.4%) were the best predictors of local recurrence. Week 3 was the optimal time point for assessing DWI imaging response. Using a combination of ∆ADC_mean_ and ∆MTV improved the strength of correlation to local recurrence (*p* ≤ 0.001). In patients who underwent both week 3 MRI and FDG-PET/CT, significant differences in local recurrence rates were seen between patients with favourable (0%), mixed (17%), and unfavourable (78%) combined imaging response.

**Conclusions:**

Changes in mid-treatment DWI and FDG-PET/CT imaging can predict treatment response and could be utilised in the design of future adaptive clinical trials.

**Clinical relevance statement:**

Our study shows the complementary information provided by two functional imaging modalities for mid-treatment response prediction in patients with head and neck cancer.

**Key Points:**

•*FDG-PET/CT and DWI MRI changes in tumour during radiotherapy in head and neck cancer can predict treatment response*.

•*Combination of FDG-PET/CT and DWI parameters improved correlation to clinical outcome*.

•*Week 3 was the optimal time point for DWI MRI imaging response assessment*.

**Supplementary information:**

The online version contains supplementary material available at 10.1007/s00330-023-09843-2.

## Introduction

Definitive radiotherapy with or without concurrent systemic treatment is a standard organ-preserving treatment for locally advanced mucosal head and neck squamous cell carcinoma. Following conventional treatment, a proportion of patients still experience locoregional tumour recurrence causing significant morbidity or death. Further, in those who are cured, the treatment is associated with significant acute and late toxicities. Commonly used tumour stage (TNM), smoking history, and HPV status provide prognostic information but have failed to be useful as predictive markers in risk-adaptation studies [1–3]. A reliable quantitative biomarker that can predict outcomes is therefore needed to guide adapted intensification or de-intensification of treatment based on response.

Functional imaging can characterise tumour biology using quantitative measures that can act as an early surrogate marker for treatment response. Diffusion-weighted imaging MRI (DWI) and 18F-fluorodeoxyglucose-positron emission tomography (FDG-PET/CT) have the advantage of non-invasively monitoring the tumour changes via serial imaging during radiotherapy. Correlational data suggest that DWI and FDG-PET/CT provide complementary biological information [4, 5]. To date, most imaging biomarker studies in head and neck cancer have analysed pre-treatment images. Changes in functional imaging performed during treatment have been shown to be a better biomarker of treatment response compared to pre-treatment imaging [6–10]. Studies analysing mid-treatment images have been limited by utilising single imaging modality, small sample size, and non-uniform methodologies. Timing of mid-treatment imaging also significantly impacts response assessment but the optimal time point for assessing response also remains unanswered.

Therefore, there remains a need for a prospective multimodality imaging study to assess the utility of quantifying mid-treatment tumour changes that predict response to radiotherapy in head and neck cancer.

The aim of this study was to evaluate if tumour changes measured using a combination of DWI and FDG-PET/CT performed serially during radiotherapy in head and neck cancer can be used to predict for treatment response. We also sought to find the optimal time point for assessing DWI in future imaging biomarker studies.

## Materials and methods

### Study design

Patients with newly diagnosed, biopsy-proven, non-metastatic mucosal head and neck squamous cell carcinoma treated with definitive radiotherapy with or without concurrent systemic therapy from two independent prospective quantitative imaging biomarker studies were evaluated. Patients in study 1 were recruited from June 2014 to May 2015. Patients in the subsequent study 2 were recruited from May 2016 to October 2019 [11]. Local research ethics committee approval was provided for both studies and all patients provided written informed consent.

All patients were evaluated and reviewed by a multidisciplinary head and neck team. Patients were treated using a simultaneous integrated boost intensity-modulated radiation therapy technique over 35 fractions. Radiotherapy treatment volumes were defined using consensus international guidelines and underwent a stringent peer review process [12].

**Fig. 1:**
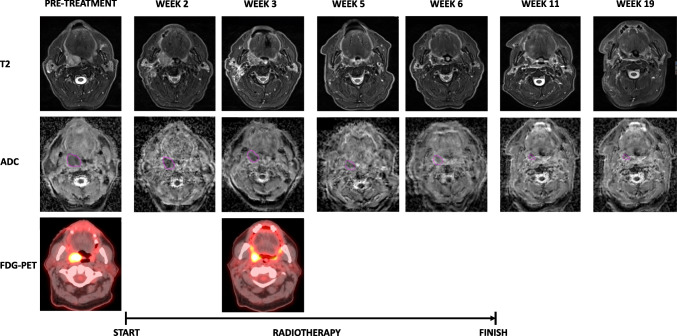
Imaging timeline and trial schema

Patients in both studies underwent serial multiparametric MRI (DWI, T2) imaging before, during (weeks 2, 3, 5, 6), and following completion (1 month and 3 months) of radiotherapy. Patients also underwent 18F-FDG-PET/CT before and during (week 3) radiotherapy. The imaging timeline utilised in both studies is shown in Fig. 1. Treatment response was evaluated with post-treatment 18F-FDG-PET/CT and clinical examination including nasoendoscopy. Recurrences were confirmed histologically or via imaging following discussion at a multidisciplinary head and neck meeting.

The imaging results were compared with the 1-year local (primary tumour) recurrence measured from time of diagnosis. Secondary outcomes included 1-year regional (nodal) recurrence, distant (non-regional nodes and visceral metastasis) recurrence, and death.

### Image acquisition

MRI was performed on a dedicated 3.0-T scanner (MAGNETOM® Skyra; Siemens Healthcare). Sequences obtained were DWI, using a readout-segmented EPI technique (RESOLVE; Siemens Healthineers), using *b* = 50 and 800 s mm^−2^ with signal averages set to 1 and 3, respectively. Morphological axial T2-weighted and T1-weighted images were also acquired. Details of the imaging technique have been described previously [11].

PET studies were acquired in radiotherapy treatment position on a GE Discovery™-710 time-of-flight positron emission tomography (PET)-CT (GE Healthcare, Waukesha, MI). FDG-PET/CT imaging was performed in accordance with EANM clinical guidelines [13]. Patients received 4.1 MBq kg^−1^ of [^18^F]FDG after at least 4 h of fasting. The average blood sugar level was 5.7 ± 1.2 mmol/L (range 3.3–9.6 mmol/L). The staging and all sequential scans were performed on the same scanner with the same acquisition and reconstruction protocols. Details of the imaging technique have been described previously [6].

### Image analysis

The primary region of interest (ROI) captured was the primary tumour. To aid accurate delineation of tumour, clinical examination and nasoendoscopic findings were provided in conjunction with patient images. All PET and MRI images were viewed and had ROIs delineated using commercial image visualisation and delineation software (MIM Software Inc.). DICOM images containing ROIs were subsequently analysed using open-source PyRadiomics software (v2.2.0) [14].

The tumour volumes were delineated by a radiation oncologist (Y.T.) in consensus with a consultant radiologist (D.M.) and nuclear medicine physician (P.L.) for each imaging modality at every time point. Due to spatially non-linear distortions associated with DWI, ROIs were manually defined on ADCmaps while primarily referencing to the co-registered low *b*-value diffusion-weighted images (b50) as per consensus recommendations and methodology performed previously [10, 15]. Co-registered T2-weighted images were also viewed at the same time to aid delineation. The mean values of apparent diffusion coefficient (ADC_mean_) were calculated from all ROIs. FDG-PET-derived metabolic tumour volume (MTV) was calculated using the PETedge tool of MIM software, a semi-automated gradient method. Other PET parameters including SUV_max_, SUV_mean_, and total lesion glycolysis (TLG = SUV_mean_ × MTV) were also calculated for all ROIs. We have previously found anatomical tumour volumes on T2 MRI and CT images and their relative change were not correlated to clinical outcomes; hence, they were not included in this analysis.

The percentage change (∆) in ADC and FDG-PET/CT imaging parameters from baseline was calculated for each time point, defined as ∆ = (value − baseline) / baseline × 100%.

### Statistical analysis

The changes in PET and DWI parameters from baseline were compared using the Wilcoxon signed-rank test.

The absolute value and change (*D*) in parameters were compared to treatment outcomes using the Mann-Whitney *U* test. For parameters with predictive value, receiver operating characteristic (ROC) analysis was performed using the area under the curve (AUC) as an index of accuracy to differentiate between multiple predictive parameters. Optimal cut-off values for analysis were derived from the ROC curves aiming for best sensitivity and specificity by applying the Youden index [16]. Local recurrence-free survival (LRFS), regional recurrence-free survival (RFS), distant metastatic recurrence-free survival (DMRFS), and overall survival (OS) curves were estimated using Kaplan-Meier analysis and compared using the log-rank (Mantel-Cox) test.

#### Combined modality response

To determine the utility of combining multiple imaging modalities, the most accurate FDG-PET/CT parameter and DWI parameter that correlated to local recurrence (highest ROC value in AUC analysis) were chosen for subsequent analysis. Optimum cut-off values from the two parameters were used to determine patient’s response in each imaging modality. Patients were subsequently divided into three groups based on those that had favourable response in both FDG-PET/CT and DWI imaging; mixed response (good PET and poor DWI, or poor PET and good DWI); or poor response in both imaging modalities (for a representative example of three groups, see Fig. 4). The three groups were compared to local recurrence status using the Pearson chi-squared test, using Cramer’s *V* test to check the strength of association to determine the utility of combining multiple parameters in improving predictive ability.

A pre-specified split-sample internal validation using the two independent enrolling studies was undertaken to explore the stability of results in combining multimodality parameters (described in detail in Supplementary Material).

The data were analysed using SPSS statistical software (version 24.0; IBM Corp). Statistical significance was considered *p* < 0.05.

## Results

### Patient characteristics

Fifty-five patients were available for analysis: 30 patients in study 1 and 25 patients in study 2. Patient, tumour, and treatment details are summarised in Table 1. The median follow-up was 34.0 months (range 4–68), with a minimum follow-up of 12 months for patients who were alive.

**Table 1:** Patient characteristics

			Study 1	Study 2	Combined total
Age at diagnosis (median)			60.8 (44–80)	63.6 (43–83)	61.4 (43–82)
Gender	Male		26	23	49 (89.1%)
	Female		4	2	6 (10.9%)
ECOG	0		20	13	33 (60.0%)
	1		9	12	21 (38.2%)
	2		1	0	1 (1.8%)
Smoker	No		7	7	14 (25.5%)
	Yes		23	18	41 (74.5%)
ETOH	Nil		8	5	13 (23.6%)
	Yes		19	16	35 (63.6%)
	Previous		3	4	7 (12.7%)
Primary tumour site	Oropharynx		19	18	37 (67.3%)
	Hypopharynx		3	4	7 (12.7%)
	Larynx		4	3	7 (12.7%)
	Nasopharynx		4	0	4 (7.3%)
T stage	T1		3	1	4 (7.3%)
	T2		14	8	22 (40.0%)
	T3		10	14	24 (43.6%)
	T4		3	2	5 (9.1%)
N stage	N0		6	4	10 (18.2%)
	N1		5	5	10 (18.2%)
	N2A		2	2	4 (7.3%)
	N2B		10	8	18 (32.7%)
	N2C		4	6	10 (18.2%)
	N3		3	0	3 (5.5%)
TNM stage	Stage 2		4	2	6 (10.9%)
	Stage 3		8	6	14 (25.5%)
	Stage 4		18	17	35 (63.6%)
P16 status	Negative		2	4	6 (10.9%)
	Positive		8	12	20 (36.4%)
	Unknown		20	9	29 (52.7%)
Grade	Well differentiated		1	1	2 (3.6%)
	Mod differentiated		4	6	10 (18.2%)
	Poor differentiated		7	11	18 (32.7%)
	Unknown		18	7	25 (45.5%)
Follow-up (months)			54.7	22.8	34.0 (4–68)
Treatment	Radiotherapy alone		5	4	9 (16.4%)
	Radiotherapy + cisplatin		22	18	40 (72.7%)
	Radiotherapy + cetuximab		3	1	4 (7.3%)
	Radiotherapy + carboplatin		0	2	2 (3.6%)
Total					**55**

The patients in two studies were not compared for differences between the groups

The 1-year local, regional, and distant recurrence rates were 18.2% (10/55), 7.3% (4/55), and 12.7% (7/55), respectively. At the time of analysis, 18 patients had died, 13 from recurrent cancer and 5 from other causes.

### Primary tumour analysis

**Fig. 2:**
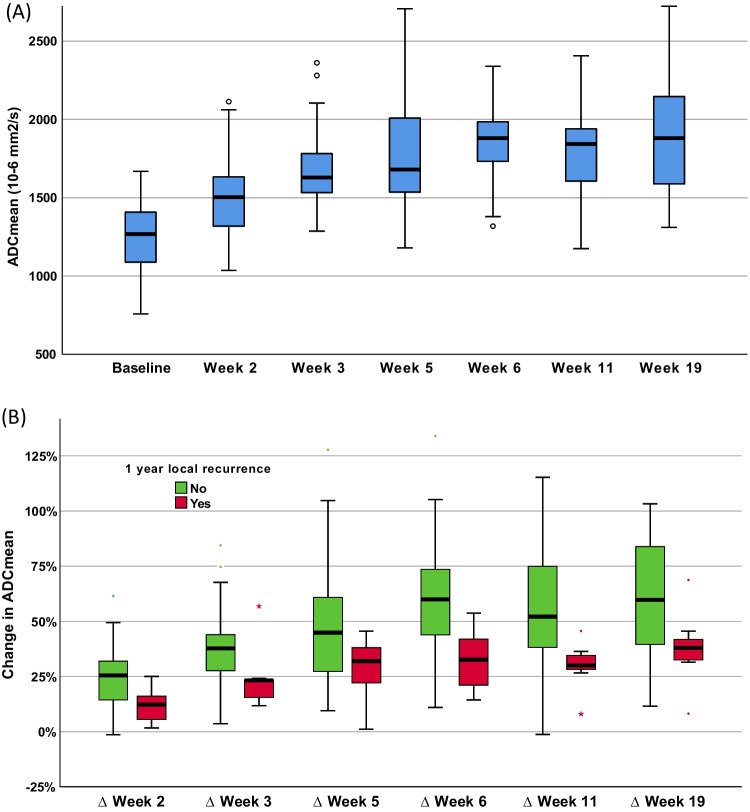
Primary tumour ADCmean values before, during, and following completion of radiotherapy. Absolute values (**A**). Percentage change relative to baseline stratified by local recurrence status (**B**)

**Fig. 3:**
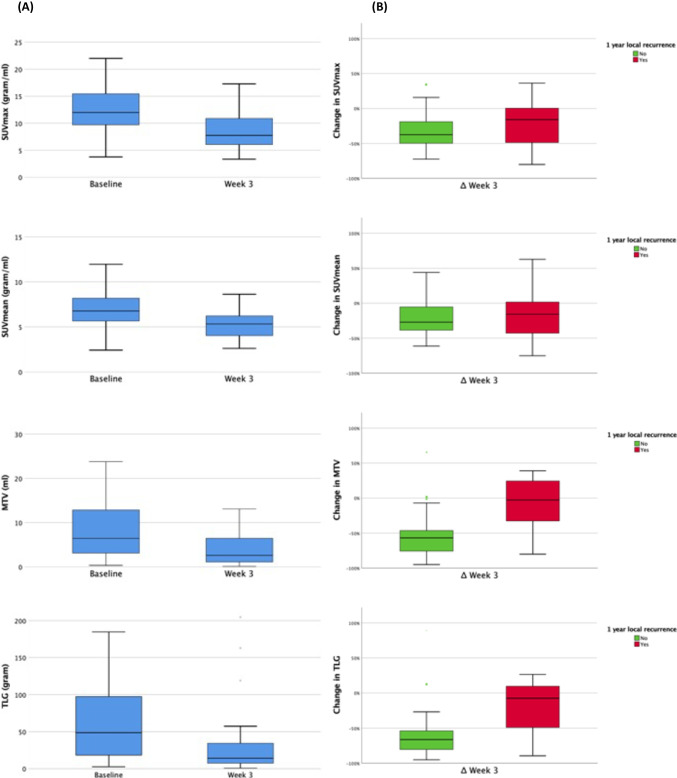
Primary tumour FDG-PET-derived parameter values before and week 3 during radiotherapy. Absolute values (**A**). Percentage change in values relative to baseline stratified by local recurrence status (**B**).

The number of DWI tumour ROIs analysed at individual time points were 55(baseline), 47 (week 2), 42 (week 3), 37 (week 5), 34 (week 6), 36 (11 weeks), and 38 (19 weeks). The number of FDG-PET/CT tumour ROIs analysed at individual time points were 55 (baseline) and 53 (week 3). The mean of primary tumour ADC_mean_ increased during radiotherapy and plateaued at week 5 (see Fig. 2A). For the entire population, the mean FDG-PET/CT parameters reduced at week 3 compared to baseline (see Fig. 3A). There was a statistically significant difference in the absolute ADC_mean_ and FDG-PET/CT parameter values at each time point relative to baseline.

### Correlation of primary tumour parameters to local recurrence

Patients with local recurrence had a higher ADC_mean_ value at baseline compared to those without. However, this did not reach statistical significance (*p* = 0.135). Absolute values of ADC_mean_ at all time points did not correlate to local recurrence. Patients with local recurrence had a lower rise in ADC_mean_ value over time (Supplementary Figure 1). The relative change in ADC_mean_ (∆ADC_mean_) at all time points except week 5 correlated to local recurrence (see Fig. 2B). The differences in the mean of absolute ADC_mean_ values and the ∆ADC_mean_ based on local recurrence status for the entire group are provided in Table 2. Relative change in ADC_mean_ at week 3 (∆Week 3 ADC_mean_) was the best predictor of local recurrence (AUC 0.825, *p* = 0.003) (Supplementary Figure 2). Optimal cut-off values of ∆Week 3 ADC_mean_ for predicting local recurrence was < 24.4% rise in ADC_mean_, resulting in 89% sensitivity, 88% specificity, PPV 97%, and NPV 67%.

**Table 2:** Comparison of primary tumour DWI and FDG-PET parameters between patients with local recurrence vs nil local recurrence at 1 year

Parameter	Local recurrence (mean, SD)	Nil local recurrence (mean, SD)	*p* value*	ROC (AUC)
SUV_max_ (g/mL)				
Baseline	14.4 ± 7.4	12.7 ± 4.0	0.266	
Week 3	9.8 ± 3.9	8.1 ± 2.9	0.228	
∆Week 3	−21.0% ± 31.8%	−34.0% ± 23.3%	0.296	
SUV_mean_ (g/mL)				
Baseline	7.3 ± 2.8	7.1 ± 2.1	0.337	
Week 3	4.5 ± 1.8	5.4 ± 1.7	0.946	
∆Week 3	−14.8% ± 36.9%	−21.4% ± 23.3%	0.982	
MTV (mL)				
Baseline	13.0 ± 11.2	10.0 ± 11.9	0.169	
Week 3	10.2 ± 7.9	4.8 ± 9.4	0.012†	0.756
∆Week 3	−5.7% ± 36.7%	−55.2% ± 29.3%	0.001†	0.833
TLG (g)				
Baseline	116.2 ± 134.3	71.5 ± 87.1	0.198	
Week 3	57.7 ± 53.3	22.8 ± 34.4	0.016†	0.747
∆Week 3	−17.3% ± 40.7%	−64.4% ± 29.6%	0.005†	0.788
ADC_mean_ (× 10^−6^mm^2^s^−1^)				
Baseline	1332 ± 170	1239 ± 222	0.183	
Week 2	1469 ± 184	1507 ± 241	0.725	
∆Week 2	12.7% ± 8.2%	23.9% ± 14.2%	0.019†	0.754
Week 3	1596 ± 106	1690 ± 263	0.946	
∆Week 3	23.3% ± 13.4%	39.1% ± 17.2%	0.003†	0.825
Week 5	1633 ± 190	1814 ± 368	0.229	
∆Week 5	28.4% ± 14.9%	48.4% ± 29.7%	0.074	
Week 6	1690 ± 335	1892 ± 325	0.058	
∆Week 6	32.3% ± 14.2%	60.8% ± 24.9%	0.005†	0.807
Week 11	1667± 213	1861 ± 350	0.068	
∆Week 11	29.9% ± 10.6%	54.2% ± 29.3%	0.011†	0.794
Week 19	1736 ± 197	1961 ± 439	0.221	
∆Week 19	37.6% ± 18.0%	61.5% ± 31.6%	0.036†	0.767

*Parameters compared to local recurrence status using Mann-Whitney *U* test

∆parameter = (mid-treatment − baseline) / baseline × 100%

†Significant (*p* < 0.05)

*ROC*, receiver operating characteristic

No absolute values of SUV_max_, SUV_mean_, MTV, and TLG at baseline correlated to local recurrence. ∆MTV (*p* = 0.001) and closely related ∆TLG (*p* = 0.005) correlated to local recurrence (see Fig. 3B). Difference in absolute values and relative change in FDG-PET/CT parameters based on local recurrence status are provided in Table 2. Relative change in MTV at week 3 (∆MTV) was the best predictor of local recurrence (AUC 0.833, *p* = 0.001). Optimal cut-off of ∆MTV for predicting local recurrence was < 50.4% drop in MTV; resulting in 91% sensitivity, 64% specificity, PPV 64%, and NPV 96%.

**Fig. 4:**
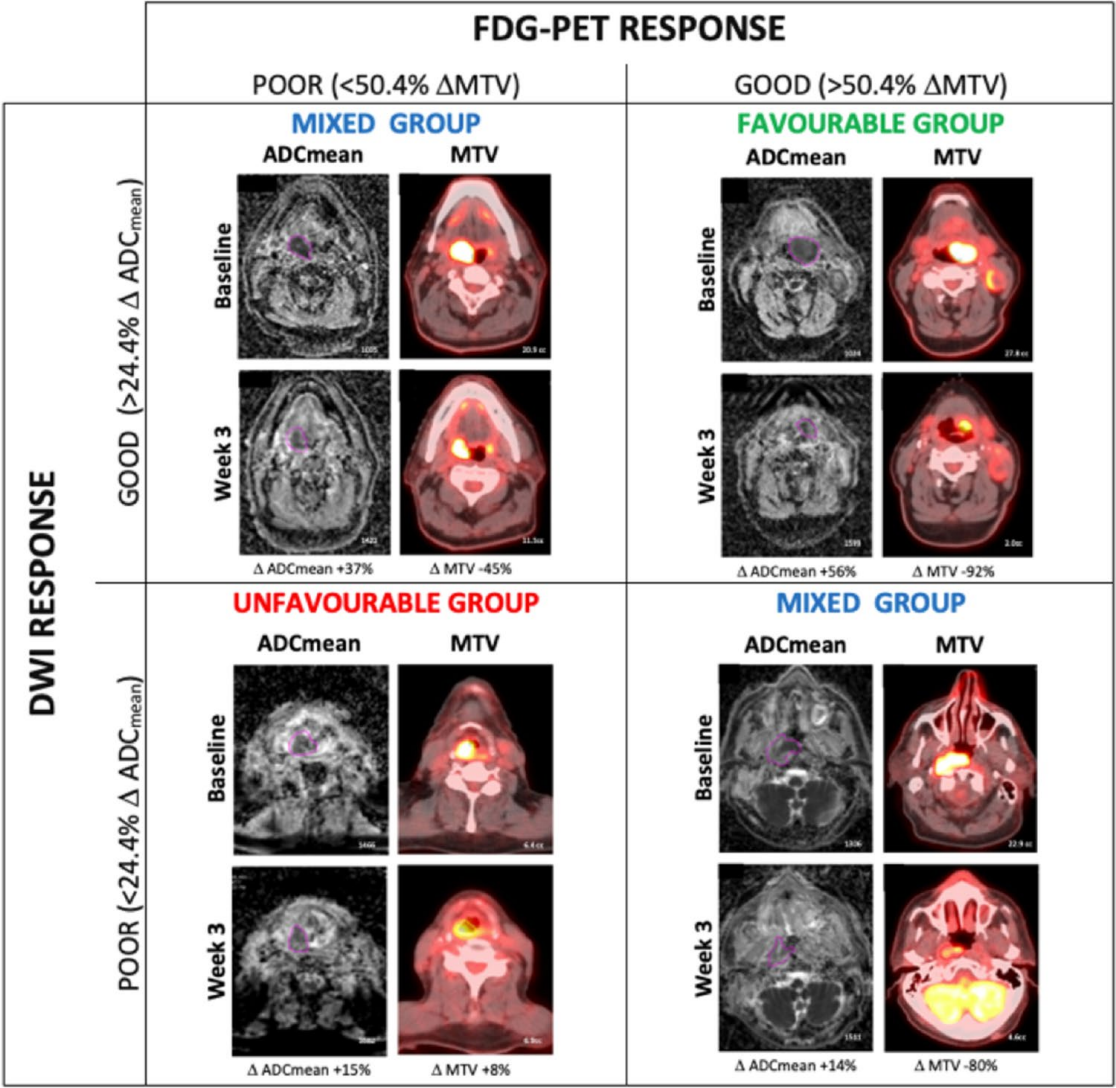
Example of patients divided into three groups (favourable, mixed, and unfavourable) based on their primary tumour response in week 3 DWI and FDG-PET imaging–derived parameters (ADC_mean_; metabolic tumour volume, MTV)

**Fig. 5:**
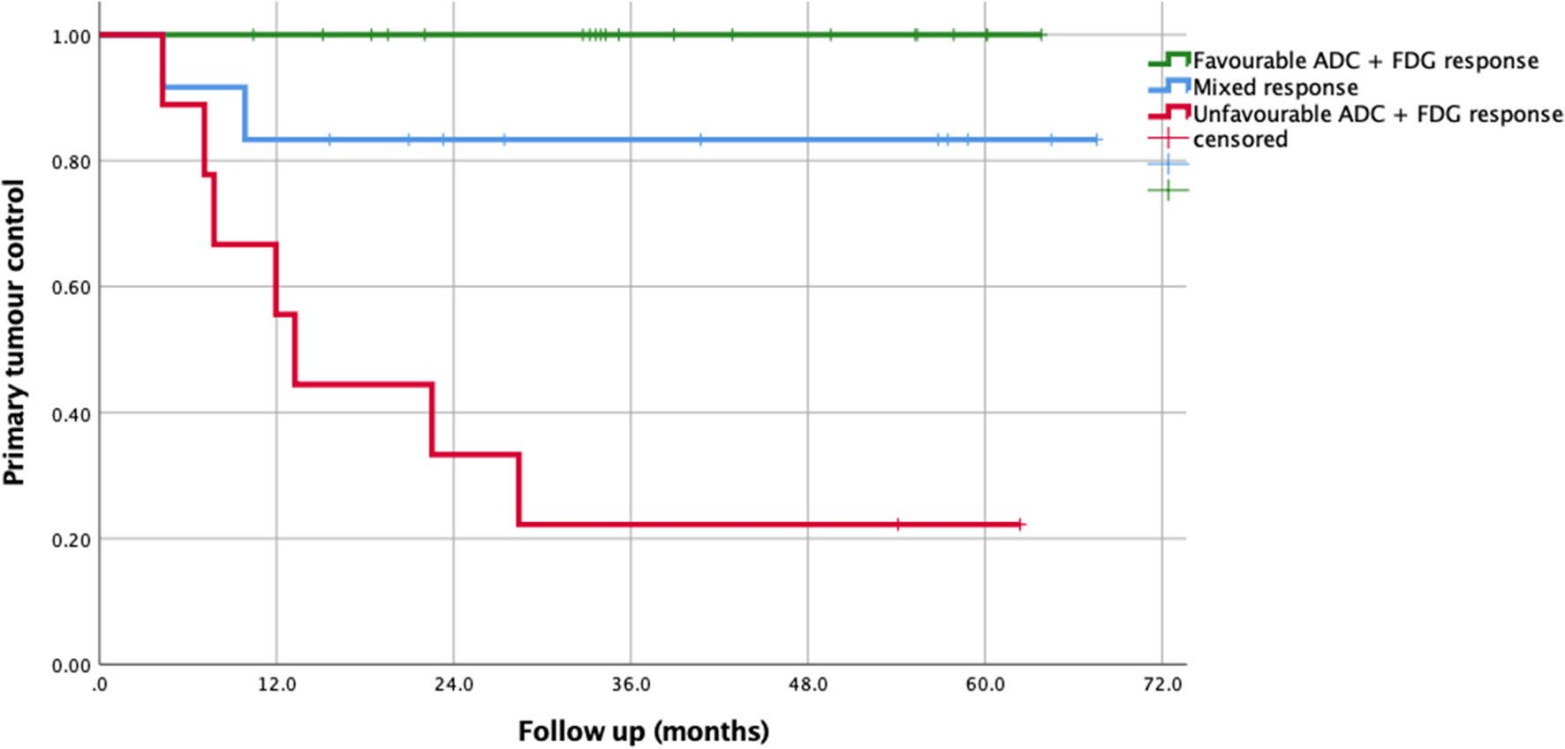
Kaplan-Meier curve for local control stratified by response in primary tumour ADCmean (> 24.4%) and FDG-PET-derived MTV (> 50.4%) at week 3 during radiotherapy

To test the primary hypothesis of the added value of combining DWI and FDG-PET/CT parameters, ∆ADC_mean_ and ∆MTV at week 3 were chosen to define response in DWI and FDG-PET/CT imaging. Using optimal cut-off value of ∆Week 3 ADC_mean_ (> 24.4%) and ∆MTV (< 50.4%) to define response in each imaging modality, 20 patients were defined as favourable responders (> 24.4% ∆Week 3 ADC_mean_, and > 50.4% ∆MTV), 9 patients were defined as unfavourable responders (< 24.4% ∆Week 3 ADC_mean_, and <50.4% ∆MTV), and 12 patients were defined as mixed responders. In favourable responders, no patients (0/20) had local recurrence. In mixed responders, 17% (2/12) had local recurrence. In unfavourable responders, 78% (7/9) had local recurrence (for a representative example of three groups, see Fig. 4). The Kaplan-Meier analysis of local recurrence stratified by the above three response groups (subgroups) is shown in Fig. 5. Using a combination of ∆ADC_mean_ and ∆MTV improved the strength of correlation to local recurrence (Cramer’s *V* 0.736, *p* ≤ 0.001, Pearson chi-squared test) compared to using individual parameter alone (Cramer’s *V* 0.693, 0.586).

As a part of pre-specified split sample internal validation, ∆Week 3 ADC_mean_ and ∆MTV were selected for analysis in patients in study 1 as a training dataset (*n* = 30). Successful internal validation was performed on the testing dataset of patients in study 2 (*n* = 25) confirming use of combined primary tumour DWI and FDG-PET/CT parameters in predicting 1-year local recurrence (see Supplementary Material for details).

### Correlation of primary tumour parameters to regional recurrence, distant recurrence, and death

Only baseline ADC_mean_ value correlated to death (AUC 0.685, *p* = 0.027) (Supplementary Table 1). Using an optimal cut-off value of 1301 × 10^−6^mm^2^s^−1^ for baseline primary tumour ADC_mean_ resulted in 2-year OS of 82% vs 65% (log rank *p* = 0.004) (see Supplementary Figure 3).

Baseline, week 3, and relative change in MTV and TLG at week 3 (∆MTV, ∆TLG) correlated to death, which occurred as a result of local tumour recurrence in majority of cases (Supplementary Table 1). Absolute value of TLG at week 3 was the best predictor for death (AUC 0.742, *p* = 0.005). Using an optimal cut-off value of 16.0 g resulted in 2-year OS of 96% vs 45%, *p* ≤ 0.001) (see Supplementary Figure 4).

## Discussion

Our study represents the largest published prospective mid-treatment imaging biomarker series utilising DWI and FDG-PET/CT scans performed during radiotherapy in head and neck squamous cell carcinoma (HNSCC). We identified functional changes in tumour during definitive radiotherapy in HNSCC using FDG-PET/CT and DWI which can be used to predict treatment response. Using a novel method, our data suggest that combination of multiple functional imaging parameters improves correlation to local tumour control and could potentially be used to identify sub-groups of patients for future risk-adapted radiotherapy clinical trials. We also identified week 3 mid-treatment as an optimal time point to perform DWI imaging to assess primary tumour response in future imaging studies.

We showed that primary tumour ADC_mean_ increased and plateaued around week 5 during radiotherapy. Change in week 3 primary tumour ADC_mean_ had the strongest correlation to local control. Based on our data, greater percentage change in ADC_mean_ (∆ > 24.4%) was highly predictive of good treatment response leading to local tumour control following radiotherapy. Studies by Motaba et al and Ghany et al also found ∆ADC_mean_ at week 3 of 24% and 20% respectively correlated to tumour control [18, 19]. The higher rise in ADC_mean_ during radiotherapy in good responders is explained by greater tumour cell loss. However, as radiotherapy progresses, increasing treatment-related oedema would also alter the ADC signal [17]. Week 3 likely represents the optimal time point for assessing treatment response due to higher ratio of signal from tumour prior to onset of noise from radiotherapy-related oedema [18]. Previous studies looking to identify the optimal time point for DWI response assessment were limited to 2 to 3 mid-treatment imaging time points [17–21]. The size of our study and extensive serial time points for DWI treatment response assessment adds considerably to the current published literature.

FDG-PET/CT imaging measures functional aspects of the tumour that are known to predict for poor radiotherapy response such as high tumour cellularity, metabolism, and/or hypoxia [19–21]. We have shown that change in primary tumour MTV at week 3 had the strongest correlation to local tumour control. Previous studies also show that FDG-PET/CT-based functional tumour volume (MTV) outperforms anatomical data for treatment response prediction [22, 23]. Based on our data, a greater percentage decrease in MTV (∆ > 50.4%) in primary tumour was highly predictive of tumour control. Our results confirm findings from previous studies that changes in FDG-PET/CT metabolic parameters are better treatment response markers compared to absolute values [22, 24]. Studies by Myo et al and Pollom et al have shown that ∆TLG from mid-treatment FDG-PET/CT can predict clinical outcomes [6, 28]. The studies utilised differing methodologies to delineate the MTV and hence limits direct comparison. Aside from non-standardised imaging protocol, performance of FDG-PET/CT-based parameters can also be limited by confounding influence of radiotherapy-induced inflammation/necrosis [24]. Hence, a rationale exists for utilisation of concomitant functional MRI imaging to provide additional discriminatory information to improve the prediction of treatment response.

We showed that utilisation of multiple functional imaging modalities improves incremental predictive performance for assessing tumour response following radiotherapy compared to individual imaging modality. FDG-PET/CT parameter provided good sensitivity but only moderate specificity due to treatment-related changes. The addition of DWI parameter improved the specificity and hence the predictive performance. The majority of published imaging biomarker studies have focused on a single imaging modality, predominantly CT, fMRI, and to a lesser extent FDG-PET and 18F-MISO PET. Multimodality functional imaging offers the advantage of measuring different tumour characteristics and improving the performance of individual imaging modalities in predicting treatment response [4, 5, 25, 26]. Wong et al have published the only other study containing 35 patients correlating changes in MRI and FDG-PET/CT imaging parameters during radiotherapy to clinical outcomes in HNSCC [10]. They found individual fMRI and FDG-PET/CT features that successfully differentiated responders from non-responders based on a surrogate endpoint of 3-month post-treatment imaging response.

Using optimal cut-off values of the highest performing FDG-PET/CT (week 3 ∆MTV) and DWI (week 3 ∆ADC_mean_) parameter, we were able to separate HNSCC patients into three risk sub-groups based on their early response during radiotherapy. This is potentially more flexible and accurate than traditional staging with AJCC as it accounts for tumour biology and treatment sensitivity. This stratification could be utilised in future mid-treatment risk-adaptation clinical trials. Patients with good FDG-PET/CT and DWI response had low rate of local failure (0%) and could be considered for de-escalation, e.g. radiotherapy dose or volume reduction, to reduce treatment-related toxicities. Patients with poor FDG-PET/CT and DWI response had high rates of local failure (78%) and could be considered for treatment intensification, e.g. radiotherapy dose boost, acceleration, use of radiosensitisers, or bail-out surgery. Our approach of risk stratifying patients is novel and hypothesis generating requiring external validation prior to clinical application. There are currently no published HNSCC studies that have utilised mid-treatment week 3 DWI and FDG-PET/CT imaging to allow external validation. Appreciating the limitation of the data available, a pre-specified split sample internal validation using patients from the two temporally separate studies was successfully undertaken to explore the stability of our results.

Strengths of our study include the large number of patients and standardised imaging time points for response assessment. Patients in our study were prospectively recruited, underwent standardised imaging protocol, and had reproducible consensus-based ROI delineation using best available guidelines [6, 10, 11, 14, 15, 27, 28]. We also correlated imaging parameters with long-term clinical outcomes rather than surrogate endpoints such as early post-treatment imaging. There are a few limitations of our study. Ours was a single-institutional study with all serial imaging undertaken on the same dedicated MRI and PET machine, and hence extrapolation of our results without additional standardisation quality assurance activities or utilisation of our methodology should be done with caution [29, 30]. We also noted attrition of patients undergoing MRI imaging as radiotherapy progressed due to poor patient tolerability; however, ∆Week 3 ADC_mean_ remained significant for assessing treatment response when analysing only patients who underwent serial DWI imaging at every time point. Patients included in our series represent the commonly seen heterogeneous sub-sites of mucosal head and neck cancer treated with radiotherapy. Therefore, our results should be replicated in a larger multi-institutional setting with a more homogeneous population prior to clinical application. In a multi-institutional setting, additional quality assurance to harmonise image quality is also required. However, we believe that parameters measuring relative change from baseline if done on the same scanner provides greater reproducibility due to auto-normalisation of measurements compared to using a single static value.

## Conclusion

Our study highlights the importance of changes in mid-treatment DWI and FDG-PET/CT imaging in predicting treatment response. We identified a combination of change in week 3 DWI (> 24.4% ∆ADC_mean_) and FDG-PET/CT (> 50.4% ∆MTV) imaging parameters performed during radiotherapy that could be utilised in the design of future risk-adapted clinical trials in HNSCC.

### Supplementary Information

ESM 1 (PDF 261 kb)

## Abbreviations

∆: Change in value = (value − baseline) / baseline × 100%
ADC_mean_: Mean value of apparent diffusion coefficient
AUC: Area under the (receiver operating characteristic) curve
DMRFS: Distant metastatic recurrence-free survival
DWI: Diffusion-weighted imaging MRI
FDG-PET: 18F-Fluorodeoxyglucose-positron emission tomography
fMRI: Functional MRI
HNSCC: Mucosal head and neck squamous cell carcinoma
LRFS: Local recurrence-free survival
MTV: Metabolic tumour volume
OC: Optimal cut-off value from receiver operating characteristic analysis
OS: Overall survival
RFS: Regional/nodal recurrence-free survival
ROI: Region of interest, primary tumour contour delineation
RT: Radiotherapy
TLG: Total lesion glycolysis
TNM: Tumour, node, and metastases staging classification

## Declarations

### Guarantor

The scientific guarantor of this publication is Dr Yuvnik Trada.

### Statistics and biometry

One of the authors has significant statistical expertise.

### Study subjects or cohorts overlap

Some study subjects or cohorts have been previously reported in initial trial protocols and preliminary analysis of technical data without clinical results.

### Methodology

•prospective

•diagnostic or prognostic study

•performed at one institution
