# Supplementary material for: Changes in serial multiparametric MRI and FDG-PET/CT functional imaging during radiation therapy can predict treatment response in patients with head and neck cancer
Source: Eur Radiol. 2023 Jul 5;33(12):8788–99. doi: 10.1007/s00330-023-09843-2 (PMC10667402; doi:10.1007/s00330-023-09843-2)
Supplement: Supplementary file 1 — (PDF 261 kb) [file 330_2023_9843_MOESM1_ESM.pdf]

Supplementary table 1: Correlation of primary tumour DWI and FDG-PET parameters with secondary outcomes (regional recurrence, distant recurrence and death).

	PRIMARY TUMOUR		
	**NR**	**DM**	**OS**
**SUV_{max} (g/mL)**			
**Baseline**	NS	NS	NS
**Week 3**	NS	NS	NS
**ΔWeek 3**	NS	NS	NS
**SUV_{mean} (g/mL)**			
**Baseline**	NS	NS	NS
**Week 3**	NS	NS	NS
**ΔWeek 3**	NS	NS	NS
**MTV (mL)**			
**Baseline**	NS	NS	0.670†
**Week 3**	NS	NS	0.740†
**ΔWeek 3**	NS	NS	0.703†
**TLG (g)**			
**Baseline**	NS	NS	0.680†
**Week 3**	NS	NS	0.742†
**ΔWeek 3**	NS	NS	0.681†
**ADC_{mean} ($\times 10^{-6} mm^2 s^{-1}$)**			
**Baseline**	NS	NS	0.685†
**Week 2**	NS	NS	NS
**ΔWeek 2**	NS	NS	NS
**Week 3**	NS	NS	NS
**ΔWeek 3**	NS	NS	NS
**Week 5**	NS	NS	NS
**ΔWeek 5**	NS	NS	NS
**Week 6**	NS	NS	NS
**ΔWeek 6**	NS	NS	NS
**Week 11**	NS	NS	NS
**ΔWeek 11**	NS	NS	NS
**Week 19**	NS	NS	NS
**ΔWeek 19**	NS	NS	NS

NR = nodal (regional) recurrence, DM = distant metastasis, OS = overall survival, LR= local recurrence
Parameters compared using Mann-Whitney U test and receiver-operator characteristics
Δ parameter = (Mid-treatment – baseline)/baseline × 100%
† Significant ($p<0.05$)

Supplementary figure 1: Trend of primary tumour ADC$_{mean}$ value before, during and following radiotherapy stratified by 1 year local recurrence status

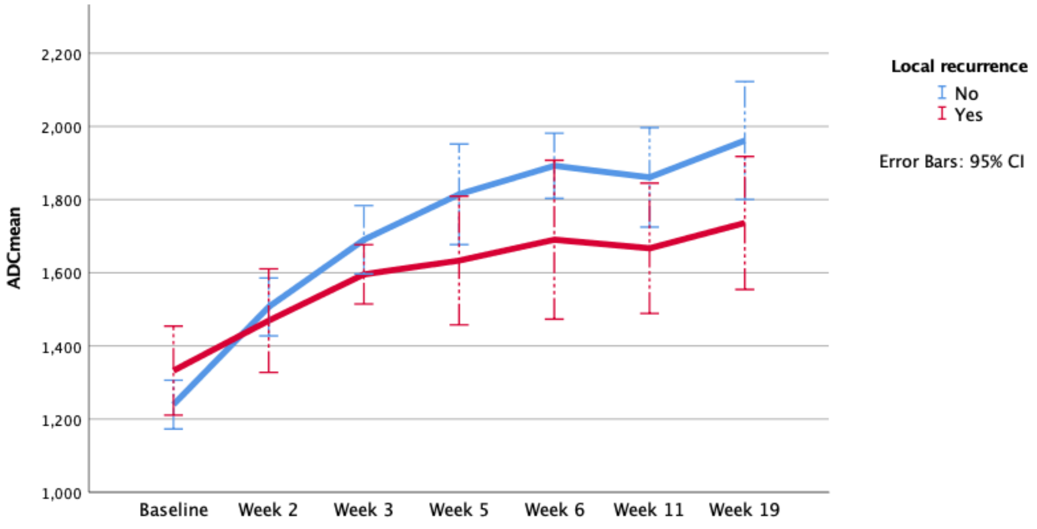

Supplementary figure 2: ROC curve for change in primary tumour ADC$_{mean}$ during and following radiotherapy correlated to local recurrence at 1 year

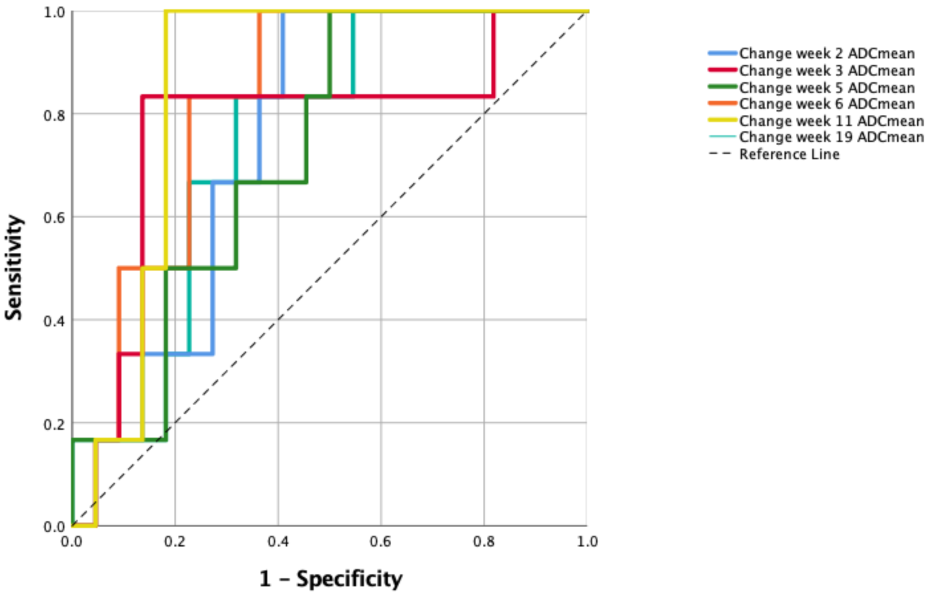

Supplementary figure 3: Kaplan Meier curve for overall survival stratified by primary tumour ADC_{mean} at baseline

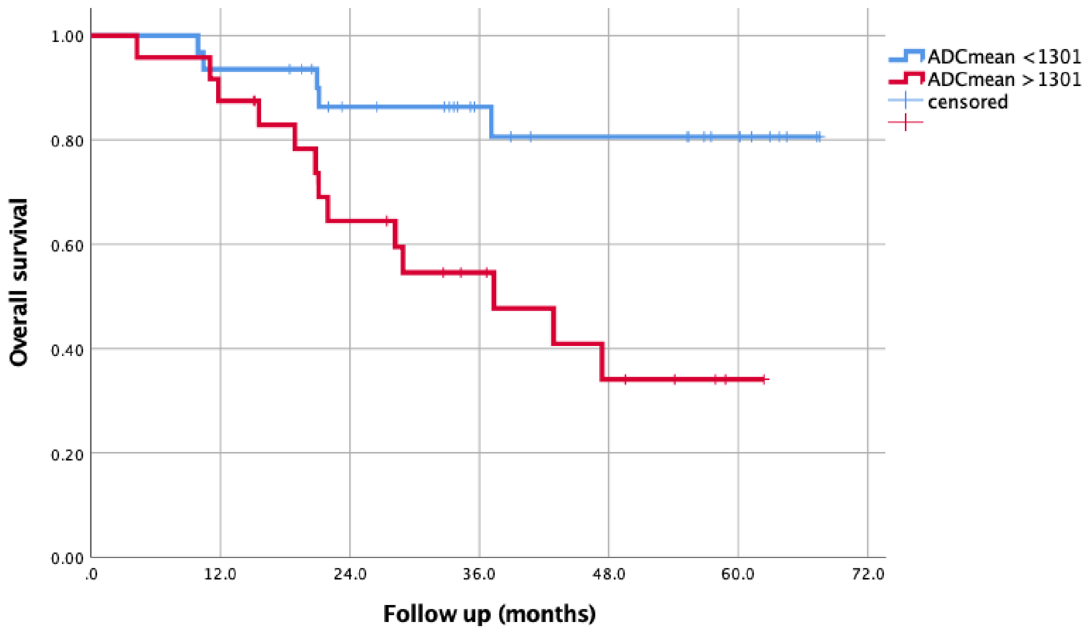

Supplementary figure 4: Kaplan Meier curve for overall survival stratified by primary tumour week 3 FDG-PET derived TLG

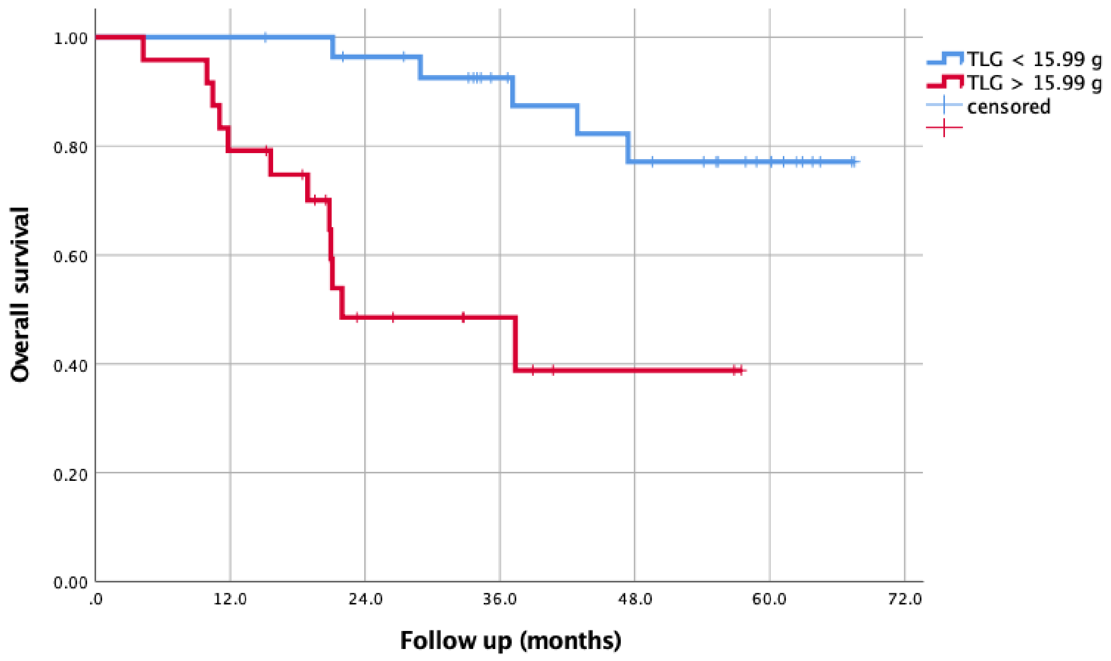

SPLIT SAMPLE INTERNAL VALIDATION

Method:

To determine the utility of combining multiple imaging modalities, the most accurate FDG-PET parameter and DWI parameter that correlated to local recurrence (highest ROC value in AUC analysis) were chosen for subsequent analysis. Optimum cut-off values from the two parameters were used to determine patient's response in each imaging modality. Patients were subsequently divided into three groups based on those that had: favourable response in both FDG-PET and DWI imaging; mixed response (good PET and poor DWI, or poor PET and good DWI); or poor response in both imaging modalities. For a representative example of three groups see Figure 5. The three groups were compared to local recurrence status using the Pearson chi-squared test, using Cramer's V test to check the strength of association to determine the utility of combining multiple parameters in improving predictive ability.

A split-sample internal validation was undertaken as an exploratory analysis to examine the results of the above approach of combining multimodality parameters. Patients were split based on the two independent studies that they were enrolled in. Study One was used as a training dataset. New optimal cut-off values were determined for both the chosen FDG-PET parameter and DWI parameter. Study Two was used as a validation dataset. Patients in the validation dataset were divided into the three groups based on the new optimal cut-off values from the training dataset, and the groups were subsequently correlated to local recurrence status using the Pearson chi-squared test.

The data were analysed using SPSS statistical software (Version 24.0; IBM Corp, Armonk, NY, USA). Statistical significance was considered as $p < 0.05$.

Results:

As a part of pre-specified split sample internal validation, ROC analysis of Δweek 3 ADC_{mean} and ΔMTV were performed using patients in Study One as a training dataset (n=30). Optimal cut-off values of primary tumour Δweek 3 ADC_{mean} (AUC 0.700) and ΔMTV (AUC 0.713) were recalculated. Patients in Study Two (n=25) were defined into three subgroups (favourable responders, unfavourable responders and mixed responders) based on new optimal cut-off values from Study One. In study 2, a statistically significant correlation between the subgroups to 1 year local recurrence was found (Pearson chi-square p=0.001, Cramer's V test 0.888), confirming an internal validation of using combined primary tumour DWI and FDG-PET parameters in predicting 1 year local recurrence.
